# Microinjection of Dihydrotestosterone as a 5*α*-Reduced Metabolite of Testosterone into CA1 Region of Hippocampus Could Improve Spatial Learning in the Adult Male Rats

**Published:** 2012

**Authors:** Soheila Babanejad, Nasser Naghdi, Seyyed Ali Haeri Rohani

**Affiliations:** a *Department of Physiology and Pharmacology, Pasteur Institute, Tehran, Iran. *; b *Department of Physiology, Faculty of Sciences, Tehran University, Tehran, Iran.*

**Keywords:** CA1, Spatial learning and memory, DHT, Androgen, Rat

## Abstract

CA1 region of hippocampus has an important role in learning and memory. Previous reports have shown that androgens like testosterone and its metabolites are present in high concentration in CA1 region of hippocampus. Androgen receptors have also high density in this region. Therefore, it is suggested that neurohormones in CA1 have an important role in learning and memory. It is likely that testosterone exerts its effect via its metabolites, especially dihydrotestosterone (DHT), a 5*α*-reduced androgen. In this research, we conducted an experiment to assess the path of testosterone›s effectiveness on spatial learning and memory. Adult male rats were randomly divided into 4 groups and, bilaterally, cannulated into CA1 region of hippocampus. One week after the surgery, animals received DMSO 0.5 μL as a control group and different doses of dihydrotestosterone (DHT) (0.25, 0.5 and 1 µg/0.5 μL/side) 25-30 min before the training in spatial version of Morris Water Maze task. Training session contained two blocks which animals had to learn the position of hidden platform in 4 trials. On the test session (next day), rats performed a one-trial probe test and then a visible platform one. The results showed that escape latency and traveled distance were decreased significantly in DHT-treated (0.5 µg/0.5 μL/side) rats. This finding suggested that DHT may have improved the effect on acquisition of spatial learning and memory.

## Introduction

Several investigations, in humans and animals, have shown the influence of androgens on learning processess using different learning tasks. There are numerous reports indicating that testosterone (T) administration can impair rat performance in spatial water maze (1-6) and inhibitory avoidance tasks (7). While some investigators have shown that the androgens improve cognitive performance (8-11), others have not been able to demonstrate a significant difference between the intact and T-depleted or T-administered male rats with respect to the spatial learning and memory (12).

Several previous findings also suggest that the metabolites of T may be responsible or mediate some of the effects on learning and memory. T is metabolized through the 5*α*-reductase enzyme to dihydrotestosterone (DHT), a nonaromatizable androgen. It was shown that T or DHT replacement in gonadectomized (GDX) rats could enhance cognitive performance (13, 14). However, DHT can be further metabolized through 3*α*-hydroxysteroid dehydrogenase (3*α*-HSD) to 5*α*-androstane, 17*β*-diol (3*α*-diol), which can also influence the cognitive performance. For example, in male rats, the administration of 3α-androstanediol (3*α*-diol) is more effective than T or DHT in improving conditioned place preference (15, 16) or, performance in spatial tasks (17), while limiting the formation of 3*α*-diol with a 3*α*-HSD inhibitor reduces the performance in the inhibitory avoidance task of intact or GDX, DHT-replaced rats compared to GDX controls (14). Although the administration of the nonaromatizable androgen, DHT, to young male rats enhances the inhibitory avoidance, it does not seem to improve the performance in the radial water maze of 22-month-old male rats (18). These data indicate that the metabolites of T may play an important role in mediating the cognition.

The hippocampus is an important target for androgens and androgens may improve cognitive performance through their action in hippocampus. The inhibitory avoidance, water maze and Y-maze tasks, are dependent upon the integrity of the hippocampus (19) and are influenced with androgen milieu (13, 20, 21). It is also known that the T-administration in GDX rats increases the hippocampal neuronal excitability (22). T-administration in male or female rats has also been shown to increase dendritic spine density in the CA3 and CA1 regions of the dorsal hippocampus and improves spatial navigation in the water maze (20, 23).

Moreover, all of the enzymes necessary for T metabolism (aromatase, 5*α*-reductase, and 3α-HSD) are located within the hippocampus (24-26) and T metabolites are readily formed in this region (24, 27, 28). These data further demonstrates that the hippocampus is a major target for T and/or its metabolites.

The study was designed to assess the effect of pre-training injections of dihydrotestosterone into the CA1 region of hippocampus on Morris water maze (MWM) performance.

## Experimental

Male albino Wistar rats (220-250 g) obtained from the Pasteur Institute of Iran were used in this study. Rats were housed in large cages (five per cage) before the surgery and individually in small cages after the surgery at room temperature of (25 ± 2°C) and standard 12:12 h light-dark cycle with light on at 07:00. Food and water were available ad-lib. These animal experimentations were carried out in accordance with the recommendations from the Declaration of Helsinki and internationally accepted principles for the use of experimental animals.

*Surgery*

Rats were anesthetized with Ketamine (100 mg/Kg IP) and xylazine (3 mg/Kg IP) and placed in a Stereotaxic instrument (Stoelting, USA). Bilateral guide cannulas were implanted in the right and left CA1 and were attached to the skull surface using dental cement and jewellers screws. Stereotaxic coordinates based on Paxinos and Watsons atlas of the rat brain were: anterior-posterior (AP), -3.8 mm from bregma; medial-lateral (ML), ± 2.2 mm from midline; and dorsal–ventral (DV), -2.7 mm from the skull surface.

*Microinjection procedure*

Intracereberal injection was done through guide cannula (23-gauge) using an injection needle (30-gauge) connected by polyethylene tubing to a 0.5 µL Hamilton micro-syringes. The injection needle was inserted 0.3 mm beyond the tip of the cannula and a 0.05 µL of vehicle (dimethyl sulfoxide, DMSO) or a different dose of dihydrotestosterone (DHT) was injected into each side of CA1 region over 2 min; the needle was left in place for an additional 60 sec before it was slowly withdrawn.R ats were divided into 4 groups that received vehicle (DMSO) or different doses of dihydrotestosterone (0.25, 0.5 and 1 µg/0.5 µL/side) 30 min before the training in MWM.

*Behavioral assessment*

*Apparatus*

The water maze is a black circular tank 136 cm in diameter and 60 cm in height. The tank was filled with water (20 ± 1°C) to a depth of 25 cm. The maze was located in a room containing many extra maze cues (*e.g*. bookshelves, refrigerator and poster). The maze was divided geographically into four quadrants [Northeast (NE), Northwest (NW), Southeast (SE), Southwest (SW)] with starting positions [North (N), South (S), West (W), East (E)] that were equally spaced around the perimeter of the tank. A hidden circular platform (diameter: 10 cm) was located in the center of the SW quadrant, submerged 1 cm below the surface of water. A video camera was mounted directly above the maze to record rat’s swimming path. A tracking system was used to measure the escape latency, traveled distance and swimming speed of each rat and also the percent of distance and the time in each quadrant.

**Figure 1:**
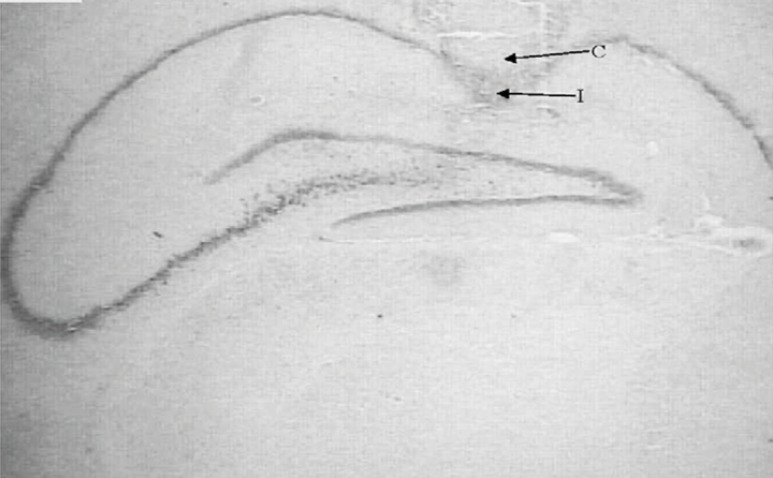
Nissl-stained coronal brain section from cannulated and injected rats. Cannula (C) and injection position (I) are shown.

*Procedure*

All testings began at 8:00 and each rat received eight trials of two blocks per day. Each rat was placed in the water facing the wall of the tank at one of the four designated starting points and allowed to swim and find the hidden platform located in the SW quadrant of the maze on every trial. Starting points were varied in a quasi-random fashion so that in each block, the rat started from each location once and never started from the same place on any block. During each trial, each rat was given 90 sec to find the hidden platform. If it found the platform, it was allowed to remain on it for 30 sec. If it failed to find the platform within 90 sec, it was placed on platform for 30 sec. During the first day, the position of platform remained constant. On the next day, the platform was elevated above the water surface and placed in the SE quadrant; this assessed Visio-motor coordination toward a visible platform.

*Histology*

Following the behavioral testing, animals were sacrificed via decapitation and their brains were removed. For the histological examination of cannula and needle placement in CA1 area (Figure 1), 100 µm thick section was taken, mounted on slides and stained with cresyl violet. The cannula track was examined for each rat. Only those animals whose cannulas were exactly placed in CA1 region (40rats) were used for data analysis.

*Experimental protocol*

The aim of this experiment was to assess the effect of pre-training injections of dihydrotestosterone into the CA1 region of hippocampus on MWM performance. Forty rats were divided into 4 groups (n = 10) and received vehicle (DMSO) or different doses of dihydrotestosterone (0.25, 0.5 and 1 µg/0.5µL/side) 30 min before the training in MWM.

**Figure 2:**
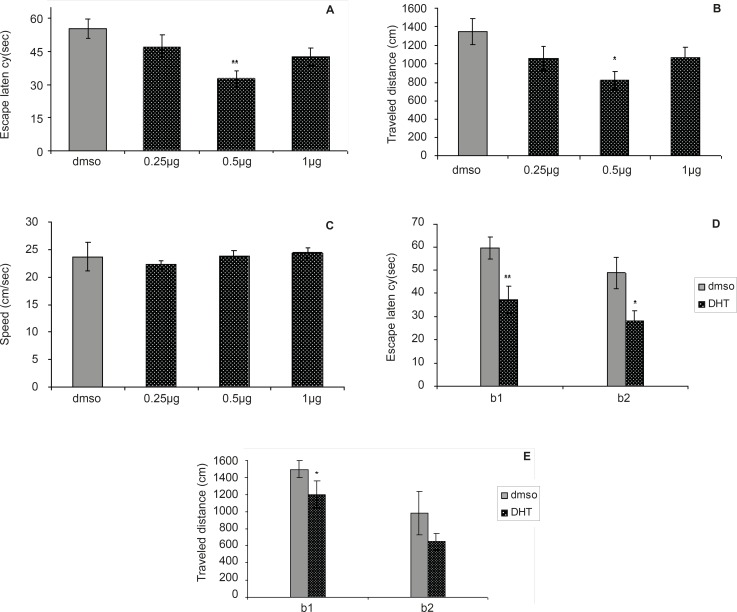
Comparison of (A) escape latency, (B) traveled distance and (C) swimming speed across training day within different groups.

**p < 0.01 and *p < 0.05 indicate significant difference vs. control (DMSO). Comparison of (D) escape latency and (E) traveled distance during each block of training day between 0.5 µg/0.5µL dihydrotestosterone treated group and the control group. **p < 0.01 and *p < 0.05 indicate the difference between each block and its respective block in control group.

*Statistical analysis*

Kolmogorov-Smirnov test was used to examine the distribution of the data. Data obtained over training days from hidden platform tests and visible platform were analyzed by t-test for comparison between two groups and one-way analysis of variance (ANOVA) followed by Tukey’s test for multiple comparisons. All results are shown as means ± SEM. In all statistical comparisons, p < 0.05 was used as the criterion for statistical significance.

## Results and Discussion

*The effect of dihydrotestosterone*

*Hidden platform trails (day 1)*

Figures 2A-2E depict the results obtained from the DHT injection and the group receiving DMSO (control). A significant difference was generally found in escape latency (F = 4.502, p = 0.0065) and traveled distances (F = 3.167, p = 0.0308) between the groups (Figure 2A and 2B). No significant difference was found in swimming speed (F = 0.3864, p = 0.7632) between groups (Figure 2C).

The differences in escape latency of block one (t = 3.227, p = 0.006) and block two (t = 2.573, p = 0.0221) and also differences in traveled distances of block one (t = 2.714, p = 0.0168) between the 0.5 µg group and control group were significantly different, but differences in traveled distances of block two (t = 2.039, p = 0.0608) were not significant (Figure 2D and 2E). This result suggests that the 0.5 µg group performed better than the control group over the training. Post-hoc multiple comparisons showed that the rats treated with 0.5 µg DHT dose had significantly improved in acquisition of spatial learning compared to the control rats.

**Figure 3:**
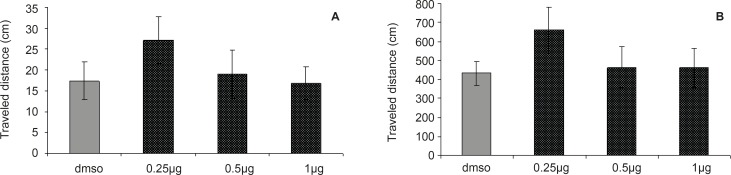
Comparison of (A) escape latency and (B) traveled distance during visible platform test. There were no significant differences between groups.

*Probe test (day 2)*

The results of probe test are shown in Figures 3A and 3B. One-way ANOVA did not reveal significant differences between the groups in escape latency (F = 0.2027; p = 0.8936) and traveled distances (F = 0.2694, p = 0.8469). The time and distance percentage that the animals spent at target quadrant was not significantly different in various groups. These results indicated that the CA1 region injection of DHT before the training was not effective in retention of memory after 24 h.

*Visible platform trials (day 2)*

**Figure 4:**
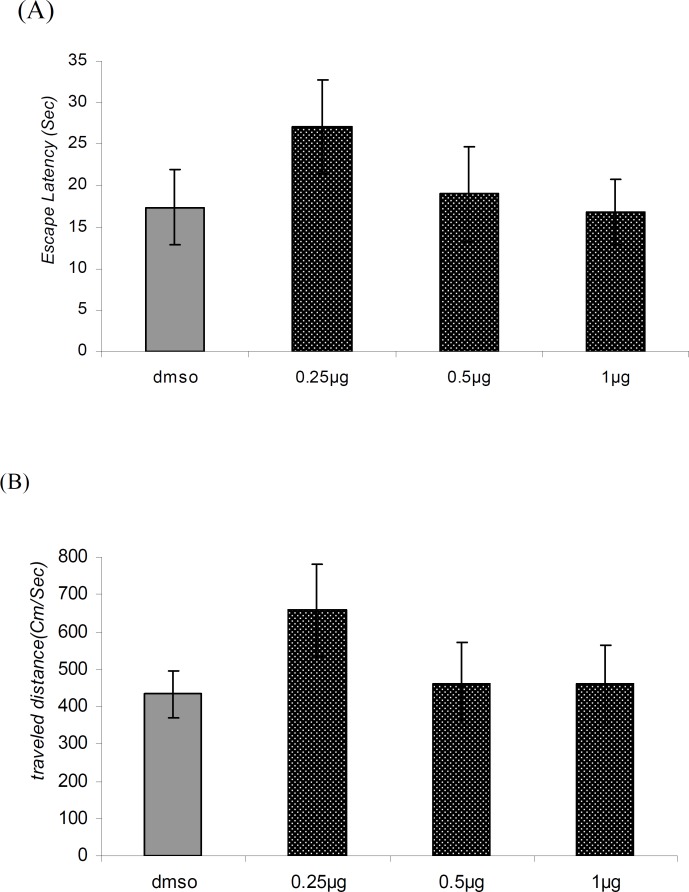
Comparison of (A) escape latency and (B) traveled distance during visible platform test. There were no significant differences between groups.

There was no significant difference in performance among the groups on the visible platform day for escape latency (F = 0.9114, p = 0.4481) or for traveled distance (F = 1.036, p = 0.3920) (Figure 4A and 4B).

In our study, adult male rats treated with different doses of dihydrotestosterone (DHT) displayed acquisition of memory improvements with a DHT dose of 0.5 µg/0.5µL in the MWM task.

Since there were no significant differences between the control and experimental groups with respect to the visible platform test, it can be inferred that the observed changes could not be attributed to the alterations of non-mnemonic factors such as motivational or sensory processes induced through the treatments.

The results of this study, along with the previous reports, conflicting effects of androgens on cognition, suggest that the cognitive/hormone interactions are quite complex. There are many researches on the cognitive performance of DHT effects as 5*α*-reduced androgens. Gonadectomy (GDX) has been shown to decrease the cognitive performance of male rats (13, 29) while systemic DHT administration is effective at enhancing the cognition of GDX rats in a variety of learning tasks (16, 17, 29-33). GDX has also been shown to reduce plasma levels of DHT and decrease the testing-training crossover latencies in the inhibitory avoidance task but DHT-replacement via silastic capsules restores performance in the inhibitory avoidance task and DHT levels of GDX rats to that of control (14) and intrahippocampal administration of DHT enhances learning in the inhibitory avoidance task compared to GDX controls (34).

Biomont *et al*. showed that DHT has no mnemonic effects on the improvement of working memory in the radial-arm maze water task in aged male rats (35).

Regarding our research and others, there are some possible explanations for the improvement effect of DHT.

First of all, there are many substrates where androgens may exert their effects to enhance cognitive performance. For instance, DHT binds with high affinity to androgen receptors (ARs) (15). DHT, like other androgens, can diffuse into the cell and bind with an intracellular cytosolic receptor, the androgen receptor (AR). This complex is then translocated to the cell nucleus where it activates the transcription of genes with androgen-responsive elements (ARE) in their promoters (36). Intrahippocampal administration of flutamide, an AR antagonist, can also reverse the beneficial cognitive-enhancing effects of DHT replacement in GDX male rats (37). Several reports suggest that in addition to the well-known traditional effects of androgens via the intracellular receptors (genomic receptors), there are nongenomic androgen receptors that have activational effects. These receptors maybe coupled to membrane ion channels and second messenger systems, which elicit the rapid and transient changes in neuronal excitability (22, 38, 39). Therefore, DHT as an androgen may also exert its mnemonic effects via genomic and nongenomic pathway.

Second, DHT is metabolized through 3*α*-hydroxysteroid dehydrogenase (3*α*-HSD) to 5*α*-androstane, 3α, 17*β*-diol (3*α*-diol). It is shown that Systemic 3*α*-diol administrations are effective at the enhancing cognition of GDX rats (13, 16, 32). In Ovariectomized rats injection of T, DHT, or 5*α* -androstane-3*α*, 17 beta-diol (3*α*-Diol) also enhanced cognitive performance in Y maze, inhibitory avoidance and object recognition tasks (17). Hippocampally administration of T and/or its 5*α*-reduced metabolites, DHT and 3*α*-androstanediol (3alpha-diol), in the conditioned fear and inhibitory avoidance tasks in intact and gonadectomized (GDX) androgen-replaced rats, showed that androgens enhancing effects on learning may be mediated in part by actions of 5alpha-reduced metabolites in the hippocampus (29-31, 33). However, 3*α*-diol was consistently elevated in each of these groups (31).

Systemic or intrahippocampal administration of 3*α*-diol increased the crossover latencies in inhibitory avoidance task and significantly, enhanced the cognitive performance as compared to the vehicle-administered in GDX rats (34). Furthermore, blocking the metabolism of DHT to 3*α*-diol through indomethacin decreased the cognitive enhancing effects of DHT in male rats (14). Therefore, it is suggested that the converting of DHT to 3*α*-diol could possibly explain the effect of DHT on learning and memory.

Third, there are multiple possible substrates for 3*α*-diols’ actions. All of the androgen administration regimens employed decrease the function of GABA_A _receptors (GBRs) in the cortex and hippocampus suggesting that GBRs is a substrate for androgen action (33). While 3α-diol has a low affinity for ARs, it has been shown to bind to GBRs (36, 40, 41), which has also been localized to the hippocampus (42), with a high affinity. 3*α*-diol, which may have effects at GABA_A _receptors (GBRs) (36, 43) can enhance learning and memory independent of T and DHT (31). Therefore, the actions at GABA_A_ receptors, for which the 3*α*-diol has a high affinity (36), may be important in mediating the androgens’ mnemonic effects.

As for the fourth point, some studies have demonstrated that the androgens like DHT may have actions at N-methyl-D-aspartate receptors )NMDARs( (44), which have been localized to the hippocampus Yoneda (45). NMDA receptor subunits (NR1, NR2A and NR2B) are involved in learning and memory processes (37). DHT treatment increased the spine synapses and NMDA receptor binding in the CA1 stratum orients and radiatum of the adult male rats (46). It is also possible that 3*α*-diol acts at these other substrates, such as N-methyl-D-aspartate receptors or via signal transduction pathways (47-49).

Another point to ponder is that the DHT’s effects to enhance the learning and memory may take place, in part, through its metabolism to 3*α*-diol, and its subsequent actions at estrogen receptor *β*) ER*β*( in the hippocampus (12, 31, 43). Knocking down expressions of ER*β*, were effective at decreasing learning and memory in 3*α*-diol replaced rats (34). It is important to note that, although there are several variants of ER*β* expressed in the brain (50, 51) this research did not discern between these different types (34). Among these splice variants, ER*β*1 is probably the only variant with sufficient abundance and affinity for estrogenic ligands to exert the important neural effects, although alterations in splicing following neural damage cannot yet be ruled out (52).
